# True Thymic Hyperplasia: An Extremely Rare Incidental Finding

**DOI:** 10.7759/cureus.87665

**Published:** 2025-07-10

**Authors:** Inês Salvado de Carvalho, Maria João Baldo

**Affiliations:** 1 Internal Medicine, Hospital Sousa Martins, Unidade Local de Saúde da Guarda, Guarda, PRT

**Keywords:** myasthenia gravis (mg), thymectomy, thymus, thymus hyperplasia, thymus neoplasms

## Abstract

Thymic hyperplasia is a rare and benign cause of mass in the anterior mediastinum in adults. This pathology is characterized by an increase in the size and weight of the thymus while preserving its architecture and histology. In most cases, it is asymptomatic, and routine imaging exams are used to make the diagnosis. Thymic hyperplasia can be divided into reactive and true hyperplasia based on morphological characteristics. The main differential diagnosis of this pathology is thymoma, an epithelial neoplasm of the thymus. True thymic hyperplasia is the rarest subtype, and, unlike reactive or lymphofollicular, it does not present histologically with lymphoid follicles and is not associated with autoimmune diseases, such as myasthenia gravis and Graves' disease. Here we present the case of an adult patient with thymic hyperplasia incidentally diagnosed in imaging exams. The established treatment was complete surgical resection, which is indicated as the first-line treatment. Subsequent histopathological analysis confirmed a diagnosis of true thymic hyperplasia.

## Introduction

The thymus is a lymphoid organ, situated in the anterior mediastinum, which has as its primary function the production, maturation, and differentiation of the T lymphocyte [1]. The epithelial thymic cells organize themselves in lobules, which are divided into the cortex and medulla [2].

Thymic hyperplasia is a rare condition characterized by an enlargement of the thymus due to an increase in the number of cells. Despite being the most common cause of masses in the anterior mediastinum in children, this condition is extremely rare in adults, with a peak incidence between 40 and 50 years old, and without gender predominance [3].

Thymic hyperplasia is defined as an abnormal growth of the thymus, and its etiology may be congenital or acquired. The congenital thymic hyperplasia is the result of a disturbance of the hypothalamic-hypophyseal axis. The acquired etiology is usually the consequence of a rebound effect in patients submitted to chemotherapy, cardiac surgery, or after discontinuation of oral corticosteroids [4]. In both etiologies, the thymus may present as hypo- or hyperfunctioning.

Thymic hyperplasia is classified morphologically as either true or reactive (lymphoid), and its diagnosis is most often incidental. True thymic hyperplasia is characterized by an increase in the size and weight of the thymus due to an increase in the number of thymic epithelial cells. The lymphofollicular hyperplasia is caused by hyperplastic lymph follicles within the thymus.

Clinically, patients may be asymptomatic more frequently, or they can present respiratory symptoms and/or upper vena cava syndrome resulting from the compression of the aorta or the upper vena cava, respectively. Thymic reactive hyperplasia, or follicular, may be associated with autoimmune diseases, such as myasthenia gravis and Graves’ disease. Patients with autoimmune diseases may present with a range of signs and systemic symptoms, depending on the underlying pathology.

The differential diagnosis of thymic hyperplasia is crucial. It should be considered in conjunction with other pathological conditions, including thymoma, thymolipoma, lymphoblastic lymphoma, thymic cysts, thymic carcinoid tumors, and thymic tumors of germ cell origin [5]. The recommended therapeutic approach is surgical resection, which enables a complete remission of the disease and is associated with a favorable prognosis [6].

## Case presentation

The female patient, 65 years old and autonomous in her daily activities, is followed in the internal medicine consultation because of heart failure with a preserved ejection fraction. During the internal medicine consultation, she complains of non-specific asthenia and adynamia, which have been evolving over several months. She denied weight loss, lack of appetite, fever, sweating, hemorrhage, dyspnea, and edema of the lower limbs.

Concurrently to the heart failure with fraction of preserved ejection in New York Heart Association class II, she presented as personal antecedents persistent auricular fibrillation, essential arterial hypertension, non-alcoholic hepatic steatosis without steatohepatitis, osteopenia, thyroid nodules, severe sleep obstructive apnea syndrome, and right pleural effusion of unknown etiology, submitted to pleural decortication in 2006. The patient was medicated with omeprazole 20 mg, one pill in fasting; bisoprolol 5 mg, one pill at breakfast; perindopril and indapamide 8 mg + 2.5 mg, one pill at breakfast; amiodarone 200 mg, one pill at lunch; apixaban 2.5 mg, one pill at breakfast and one pill at dinner; and zolpidem 10 mg, one pill at bedtime.

Upon objective examination, she presented with mucocutaneous paleness, with no other significant changes. Due to a prolonged state of asthenia, a complementary study was requested for neoplasia screening. We highlight laboratory normochromic normocytic anemia (hemoglobin 11.7 g/dl, mean corpuscular volume 88.6 fl, mean corpuscular hemoglobin 28.5 pg, mean corpuscular hemoglobin concentration 32.1 g/dl), iron (30 ug/dl), and index of transferrin saturation (12%) diminished with total capacity of normal iron connection, ferritin, transferrin, folic acid, and vitamin B12 (Table 1). Without any change in the thyroid function, the electrophoresis of serum proteins.

**Table 1:** Patient's laboratory values Hb: hemoglobin, MCV: mean corpuscular volume, MCH: mean corpuscular hemoglobin, MCHC: mean corpuscular hemoglobin concentration

Laboratory values	Patient values	Reference values
Hb (g/dL)	11.7	12-16
VCM (fl)	88.6	80.0-100.0
HCM (pg)	28.5	26.0-36.0
CHCM (g/dl)	32.1	31.0-37.0
Ferritin (ng/mL)	96.6	4.6-204.0
Index of transferrin saturation (%)	13	20-50
Total capacity of normal iron connection (ug/mL)	296	240.0-450.0
Transferrin (mg/mL)	265	173.0-204.0
Iron (ug/mL)	38	50-170
Folic acid (ng/mL)	15.1	3.1-20.5
Vitamin B12 (pg/mL)	521	187-883
TSH (µUI/mL)	1.018	0.350-4.960
T4 (ng/dl)	1.4	0.7-1.5
Electrophoresis of sérum proteins (g/dL)		
Albumin fraction	3.88	4.0-4.8
Alpha 1 fraction	0.37	0.20-0.40
Alpha 2 fraction	0.79	0.50-0.90
Beta 1 fraction	0.43	0.40-0.90
Beta 2 fraction	0.34	0.20-0.50
Gamma fraction	1.23	0.80-1.40

**Figure 1:**
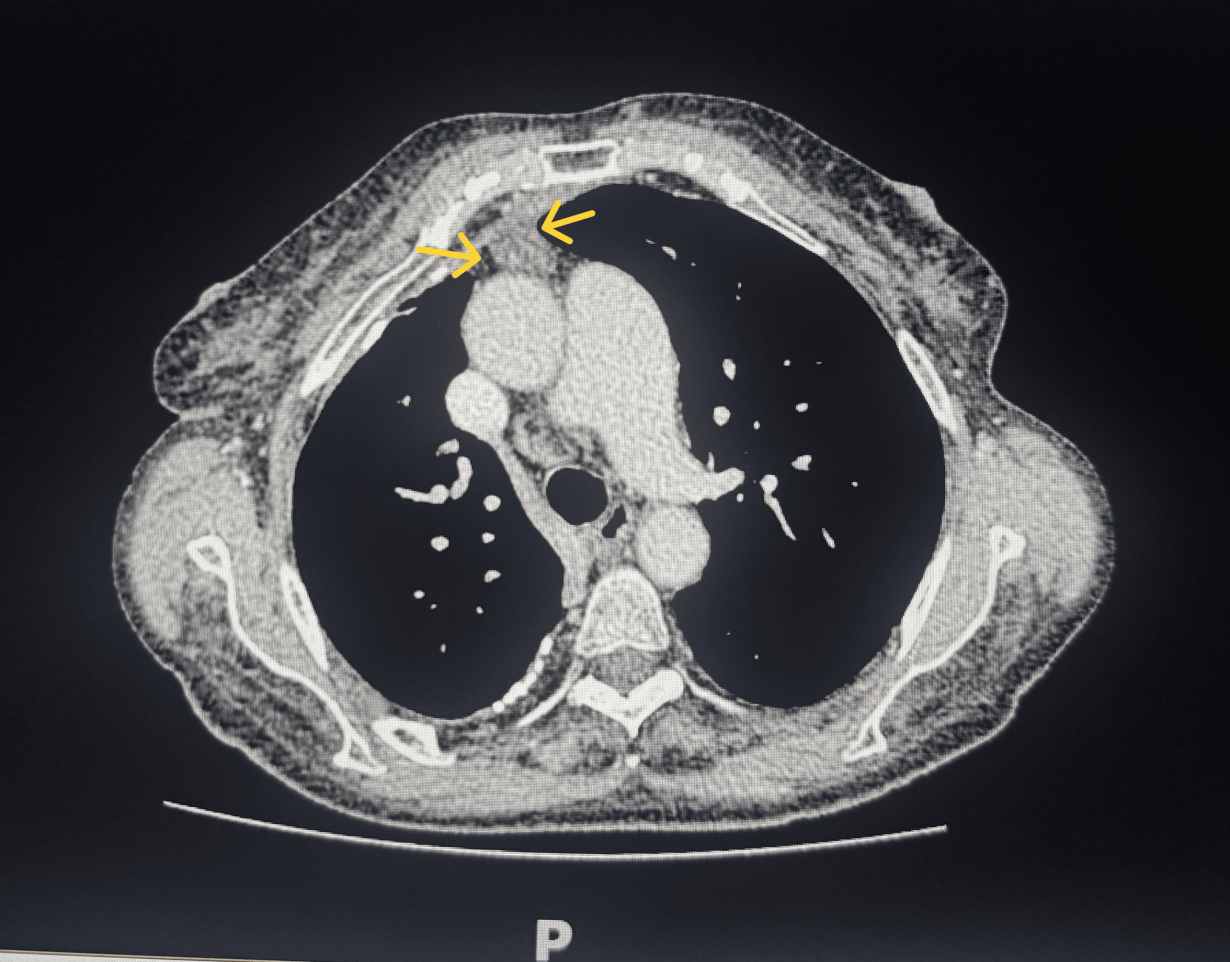
Chest CT image of the patient CT: computed tomography

**Figure 2:**
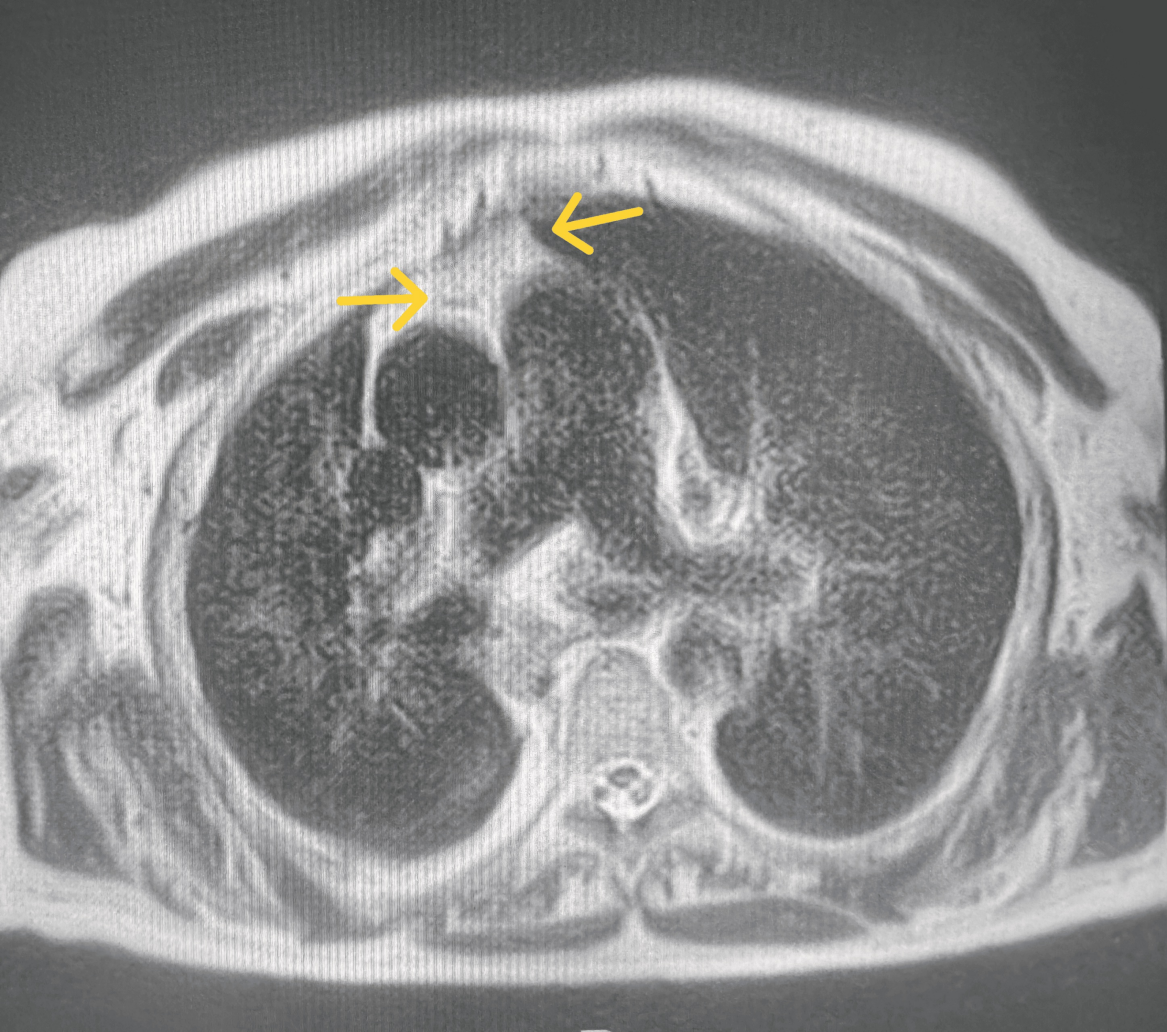
Chest MRI image of the patient MRI: magnetic resonance imaging

To make a complementary study of the clinical state, it was asked: high digestive endoscopy, which revealed an alkaline chronic gastritis; colonoscopy, which showed internal hemorrhoids of degree I/IV; and pelvic abdominal thoracic CT, which identified "calcifications in the right lung and densification of the thymic fat of the anterior mediastinum with a discrete effect of distinctness and mild pericardial thickening with calcifications" (Figure 1). To obtain a better illustration of the thymic pathology identified on the CT scan, a chest MRI was performed, which revealed an increase in the cardiothoracic index, with a right deviation of the mediastinum and dilation of the pulmonary artery. On the chest MRI, it was identified: "In the anterior mediastinum, at the pre-vascular level, an image of nodular densification, of triangular morphology, with 28 x 13 mm of bigger dimensions in the axial plane and about 40 mm of longitudinal dimension, with associated adipose infiltration, but without delimited solid nodules and local invasion behavior, probably translating to a thymoma/thymic hyperplasia" (Figure 2).

Due to the association of thymic hyperplasia with autoimmune diseases, such as Graves' disease and myasthenia, it was suggested to dose anti-receptor antibodies, acetylcholine, and anti-muscarinic (associated with Graves' myasthenia), as well as anti-thyroid peroxidase and anti-thyroglobulin (associated with Graves' disease), which presented a negative result. An electromyography was also performed with a normal result.

The patient was referred to the thoracic surgeon, where she underwent J-mini sternotomy thymectomy without any complications. Based on the subsequent anatomopathological study, it was concluded that the condition was a true thymic hyperplasia. In an internal medicine follow-up consultation, the patient was referred for the resolution of complaints of asthenia after thoracic surgery.

## Discussion

True thymic hyperplasia is characterized by a massive enlargement of the thymus, accompanied by an increase in weight and size, which increases the number of thymic epithelial cells. Nevertheless, we observe the preservation of the architecture and histological morphology of the thymus in the absence of lymphoid follicles, which distinguishes this clinical condition from reactive thymic hyperplasia [7].

As in the described clinical case, the diagnosis is often incidental, particularly when imaging tests are performed (such as CT or MRI), especially in the absence of symptoms. The association with systemic symptoms may occur, particularly when a subclinical autoimmune disease is present. The patient in the case did not present systemic symptoms that suggested an association with autoimmune disease, which was later confirmed in the requested complementary study.

Reactive thymic hyperplasia is more common and is characterized by the presence of numerous lymphoid follicles with germinal centers at both the marrow level and the interlobular space [8]. Unlike true thymic hyperplasia, this form is associated with autoimmune diseases, such as Graves' disease and myasthenia gravis. In 75% of patients with myasthenia gravis, we observe thymic disease, from which 65% present with lymphoid thymic hyperplasia and 10% with thymoma [8].

Other conditions that follow the thymic growth must also be distinguished from thymic hyperplasia, like thymoma. The thymoma is characterized by an abnormal thymus with the presence of a neoplastic focus and progresses with a more aggressive clinical evolution. The definitive diagnosis of the thymic disease is based on the histological analysis. Although controversial, the consensus of the scientific community is that histological diagnosis should be performed after surgical resection, with no need for a previous biopsy, thereby avoiding the destruction of tumor integrity and consequently its dissemination [6].

Surgical resection of the thymus gland is used to treat thymic tumors (thymoma, thymic carcinoma, neuroendocrine tumors, and thymic hyperplasia) and for the management of myasthenia gravis associated with reactive thymic hyperplasia. Thymectomy has been proven to achieve complete, stable remission.

## Conclusions

Thymus growth can have several causes, and regardless of the etiology, it presents with nonspecific symptoms, which makes diagnosis difficult. Thymic hyperplasia, primarily the true type, is a rare pathology, and there are few clinical cases described in the scientific literature. In the scientific community, several uncertainties still exist regarding the adjacent pathological process, despite recognizing that it presents a benign clinical evolution. Histological analysis after surgical resection, the recommended therapeutic approach, allows a definitive diagnosis of the disease. True thymic hyperplasia is a rare entity whose diagnosis is mostly incidental but which has a definite treatment, namely surgical resection, and has a favorable prognosis.
